# Radiotherapy quality assurance review in a multi-center randomized trial of limited-disease small cell lung cancer: the Japan Clinical Oncology Group (JCOG) trial 0202

**DOI:** 10.1186/1748-717X-4-16

**Published:** 2009-06-02

**Authors:** Naoko Sanuki-Fujimoto, Satoshi Ishikura, Kazushige Hayakawa, Kaoru Kubota, Yutaka Nishiwaki, Tomohide Tamura

**Affiliations:** 1 Clinical Trials and Practice Support Division, Center for Cancer Control and Information Services, National Cancer Center, 5-1-1 Tsukiji, Chuo-ku, Tokyo 104-0045, Japan; 2 JCOG radiotherapy committee, Clinical Trials and Practice Support Division, Center for Cancer Control and Information Services, National Cancer Center 5-1-1 Tsukiji, Chuo-ku, Tokyo 104-0045, Japan; 3 JCOG lung cancer study group, Thoracic Oncology Division, National Cancer Center Hospital, 5-1-1 Tsukiji, Chuo-ku, Tokyo 104-0045, Japan

## Abstract

**Background:**

The purpose of this study was to analyze the radiotherapy (RT) quality assurance (QA) assessment in Japan Clinical Oncology Group (JCOG) 0202, which was the first trial that required on-going RT QA review in the JCOG.

**Methods:**

JCOG 0202 was a multi-center phase III trial comparing two types of consolidation chemotherapy after concurrent chemoradiotherapy for limited-disease small cell lung cancer. RT requirements included a total dose of 45 Gy/30 fx (bis in die, BID/twice a day) without heterogeneity correction; elective nodal irradiation (ENI) of 30 Gy; at least 1 cm margin around the clinical target volume (CTV); and interfraction interval of 6 hours or longer. Dose constraints were defined in regards to the spinal cord and the lung. The QA assessment was classed as per protocol (PP), deviation acceptable (DA), violation unacceptable (VU), and incomplete/not evaluable (I/NE).

**Results:**

A total of 283 cases were accrued, of which 204 were fully evaluable, excluding 79 I/NE cases. There were 18 VU in gross tumor volume (GTV) coverage (8% of 238 evaluated); 4 VU and 23 DA in elective nodal irradiation (ENI) (2% and 9% of 243 evaluated, respectively). Some VU were observed in organs at risk (1 VU in the lung and 5 VU in the spinal cord). Overall RT compliance (PP + DA) was 92% (187 of 204 fully evaluable). Comparison between the former and latter halves of the accrued cases revealed that the number of VU and DA had decreased.

**Conclusion:**

The results of the RT QA assessment in JCOG 0202 seemed to be acceptable, providing reliable results.

## Introduction

Quality assurance (QA) and quality control are an integral part of multi-center clinical trials involving radiotherapy (RT). Several reports have shown that failure to adhere to the treatment protocol deteriorated the outcome in clinical trials [1-5]. To provide reliable results in clinical trials, it is important to keep each treatment as uniform as possible. In addition, a QA program is indispensable for patient safety, preventing increased or unexpected toxicity, and ensuring a certain effect.

In 1999, Japan Clinical Oncology Group (JCOG) trial 9812 was started to evaluate whether RT with carboplatin would result in longer survival than RT alone in elderly patients with unresectable stage III non-small cell lung cancer; however, due to excessive serious adverse events, the trial was terminated early when 46 patients were registered. By retrospective RT QA review, a protocol violation was revealed in 60% of the cases [6].

**Figure 1:**
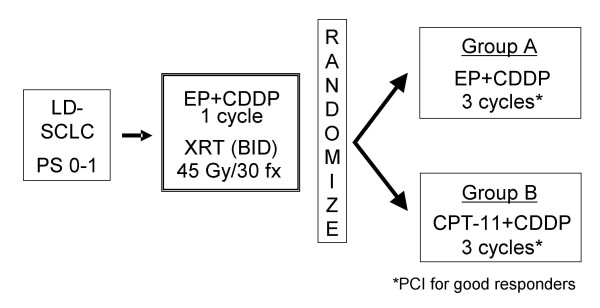
**Schema of JCOG 0202**. Abbreviations. LD-SCLC, limited-disease small cell lung cancer; PS, performance status; EP, etoposide; CDDP, cisplatin; XRT, thoracic radiotherapy; BID; bis in die/twice a day; CPT-11, irinotecan; PCI, prophylactic cranial irradiation.

JCOG 0202 was a multi-center phase III trial comparing two types of consolidation chemotherapy after concurrent chemoradiotherapy for limited-disease small cell lung cancer (Figure 1).

The primary endpoint of JCOG 0202 was overall survival and the secondary endpoints included disease-free survival and the toxicity profile of each treatment. This trial was the first in JCOG to require on-going RT QA to improve the quality of clinical trials. This is a retrospective evaluation of the protocol compliance of JCOG 0202.

## Methods

### Study design and RT requirements

After enrolling in this trial, patients received cisplatin 80 mg/m^2 ^on day 1 and etoposide 100 mg/m^2 ^on days 1–3, with concurrent RT. Patients were randomized after chemoradiotherapy and received either 3 cycles of the same chemotherapy of cisplatin and etoposide every 3 weeks, or cisplatin 60 mg/m^2 ^on day 1 and irinotecan 60 mg/m^2 ^on days 1, 8 and 15 every 4 weeks.

RT requirements included a total dose of 45 Gy in 30 fractions (bis in die, BID/twice a day) with an interfraction interval of over 6 hours. For treatment planning, both conventional 2-dimensional (2-D) X-ray simulation and 3-dimensional (3-D) CT simulation were allowed. PET scanning was not required in RT planning. Gross tumor volume (GTV) was defined as the primary tumor demonstrated by CT scan as well as metastatic lymph nodes measuring 1 cm or greater in short axis. In this trial, the clinical target volume (CTV) for the primary tumor and metastatic lymph nodes was created without adding any margins to GTV. CTV also included a regional (elecitve) nodal area which consisted of ipsilateral hilum and bilateral mediastinal (pretracheal, paratracheal, tracheobroncheal, and subcarinal) lymph nodes. Contralateral hilar lymph nodes were not included in the CTV. The planning target volume (PTV) was created by adding margins at the discretion of radiation oncologists (typically 0.5–1 cm for lateral margin and 1–2 cm for cranio-caudal margin, depending on respiratory motion and patient fixation). A dose of 30 Gy was prescribed at the center of the PTV, including elective nodal irradiation (ENI), followed by a boost dose of 15 Gy to the primary tumor and metastatic lymph nodes. Tissue heterogeneity correction was not used for monitor unit calculation, because if heterogeneity correction was required and different calculation algorithms were allowed, inter-institutional variation of the delivered dose would have been significant, and the convolution-superposition algorithm was not available in some participating institutions at the beginning of this trial.

Dose constraints were defined in regard to the dose to the spinal cord and the lung. The dose to the spinal cord was kept at ≤ 36 Gy. A posterior spinal shield was not allowed. The percentage of normal lung volume minus PTV receiving 20 Gy or greater (V_20_) was kept ≤ 35%. In 2-D planning, the field size was limited to ≤ half of the ipsilateral lung (for upper lobe tumors, ≤ 2/3).

### Quality assurance review

**Figure 2:**
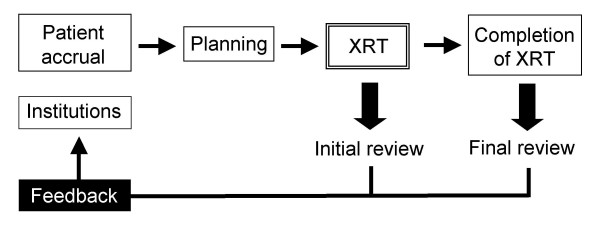
**Flow of QA review**. After the QA review, feedback was given to the institutions. Treatment planning was modified when possible.

For initial QA review, copies of pre-treatment diagnostic chest X-ray and CT, simulation and portal films, worksheets for monitor unit calculation of the prescribed dose, and RT charts with the record of the irradiated time were collected. Information on the initial RT plan was required to be sent to the QA review center within 7 days after the start of RT. Information on the total course of RT, including the boost treatment plan, was required to be sent within 30 days after completion of RT. These were reviewed periodically at least twice a month by the RT principal investigator (S.I.), and also by an independent radiation oncologist (N.S.) after patient accrual. RT QA for prophylactic cranial irradiation was not performed. After the review of the initial RT plan, the RT principal investigator sent each institution a letter reporting whether they had complied with the treatment protocol as well as an inquiry about QA documentation when necessary (Figure 2). Progress remarks and problems were reported at periodical meetings for investigators.

To assess protocol compliance for RT, the following parameters were reviewed: the dose and field border placement for PTV (adequacy of margins for GTV and ENI), doses to organs at risk, such as the spinal cord and the normal lung, overall treatment time, interfraction interval, and dose calculation without heterogeneity correction. The QA assessment was given as per protocol (PP), deviation acceptable (DA), violation unacceptable (VU), and incomplete/not evaluable (I/NE). The criteria were set for each parameter as follows. For the dose and field coverage of GTV, VU was defined as a dose less than 40.5 Gy, more than 49.5 Gy, or the distance between the field edge of the blocks or multileaf collimators and the rim of GTV less than 1 cm or more than 3.5 cm. For the dose and field coverage of ENI, a dose less than 27 Gy, more than 36 Gy or inclusion of the contralateral hilum was judged as VU. If hererogeneity correction was used for dose calculation and the recalculated uncorrected dose deviated more than 10%, it was judged as VU. Other criteria for the QA assessment are listed in Table 1. These criteria were arbitrary rather than based on the literature. We set these criteria based on the patterns of practice in Japan at the start of this trial. After parameter compliance was assessed, overall RT compliance was determined as PPoverall, no DA or VU in any parameter; VUoverall, at least one VU in any parameter; or DAoverall, neither PP nor VU. The proportion of 2-D X-ray simulation vs. 3-D CT simulation was analyzed, and a comparison was also made between compliance in the first half vs. the second.

**Table 1:** Criteria for QA scores

	PP	DA	VU
GTV			
distance to field borders	1 – 3.5 cm	NA	< 1 cm or > 3.5 cm
prescribed dose	45 Gy	Neither PP nor VU	< 40.5 Gy or > 49.5 Gy
ENI			
distance to field borders	1 – 3.5 cm	Neither PP nor VU	contralateral hilum included
prescribed dose	27 – 36 Gy	NA	< 27 Gy or > 36 Gy
Overall treatment time	21 – 42 days	NA	> 42 days
Interfraction interval	≥ 5.5 hrs	4 – 5.5 hrs or <4 hrs (once)	< 4 hrs more than once
Organs at risk			
Spinal cord	≤ 36 Gy	Neither PP nor VU	> 39 Gy
Lung	≤ 1/2 ipsilateral hemithorax (≤ 2/3, upper lobe tumor) or V_20 _≤ 35%	Neither PP nor VU	> 1/2 ipsilateral hemithorax (> 2/3, upper lobe tumor) or V_20 _> 40%
Heterogeneity correction	No	Yes (≤ 10% total dose difference)	Yes (> 10% total dose difference)

Abbreviations: PP, per protocol; DA, deviation acceptable; VU, violation unacceptable; GTV, gross tumor volume; ENI, elective nodal irradiaton; NA, not applicable; hrs, hours; V_20_, percentage of the total lung minus PTV receiving ≥ 20 Gy.

## Results

From September 2002 to September 2006, 283 cases were accrued. Of these, 204 (72%) were fully evaluable, excluding 79 cases (Table 2). Partially evaluable cases were included to evaluate each item.

**Table 2:** Number of evaluable cases and overall RT compliance

	number	(%)
Total	283	
Data insufficient/partially evaluable	62	
Off-protocol	12	
Ineligible	5	
Fully evaluable	204	(100)
PPoverall	158	(77)
DAoverall	29	(14)
VUoverall	17	(8)
Compliance (PPoverall+DAoverall)	187	(92)

Abbreviations: PP, per protocol; DA, deviation acceptable; VU, violation unacceptable

Among 258 patients evaluable for the treatment planning method, conventional 2-D X-ray simulation was performed in 62 (24%) patients, while 196 (76%) had 3-D CT simulation. Of 35 participating institutions, 24 institutions had introduced 3-D CT simulation, 6 used only 2-D X-ray simulation, and 5 used both.

RT compliance for each parameter is listed in Table 3. There were 18 VU in GTV (8% of 238 evaluated), of which, 14 (78%) had insufficient lateral margins, while 3 (17%) and 2 (11%) had insufficient caudal and cranial margins, respectively (one case, both lateral and caudal margins). There was no VU in the GTV dose. With regard to ENI, 4 VU and 23 DA (2% and 9% of 243 evaluated, respectively) were observed. Of these 4 VU, a total dose of 45 Gy instead of 30 Gy was given in 3, and the contralateral hilum was irradiated in one case. Of these 23 DA, 17 had larger field placement than required in the protocol, such as the inclusion of uninvolved supraclavicular fossa, upper mediastinum, or subaortic/paraaortic lymph node area, etc, whereas 3 had insufficient margins. Three had both larger field placement and insufficient margins. No VU was found in overall treatment time, interfraction interval and dose calculation, while some VU were observed in organs at risk (1 VU in the lung and 5 VU in the spinal cord). Overall RT compliance (PP + DA) was 92% (187 of 204 fully evaluable).

**Table 3:** RT compliance for each parameter

	Evaluable cases	PP	(%)	DA	(%)	VU	(%)
GTV	**238**	**220**	(92)	**NA**		**18**	(8)
ENI	**243**	**216**	(89)	**23**	(9)	**4**	(2)
Overall treatment time	**227**	**227**	(100)	**NA**		**0**	(0)
Interfraction interval	**205**	**195**	(95)	**10**	(5)	**0**	(0)
Organs at risk							
Spinal cord	**236**	**231**	(98)	**0**	(0)	**5**	(2)
Lung	**246**	**245**	(100)	**0**	(0)	**1**	(0.4)
Heterogeneity correction	**244**	**228**	(93)	**16**	(7)	**0**	(0)

Abbreviations: PP, per protocol; DA, deviation acceptable; VU, violation unacceptable; GTV, gross tumor volume; ENI, elective nodal irradiaton; NA, not applicable.

In regard to the 35 participating institutions, 17 (49%) had no VU. In 18 institutions with VU, 15 (83%) had only one VU and 3 (17%) had 2 or more VU. Sixteen institutions (89%) had VU in their first 3 cases.

Comparison between the former and latter halves of the accrued cases (141 and 142 cases, respectively) revealed that the number of VU and DA had decreased: for GTV, the number of VU was 13 in the early period (9%; 95% CI, 5%–15%), while 5 in the late period (4%; 95% CI, 1%–8%). In regard to ENI, DA decreased from 20 (14%; 95% CI, 9%–21%) to 3 (2%; 95% CI, 0.4%–6%), respectively.

## Discussion

In clinical trials, patients must receive optimal treatment. Since the 1980s, a number of reports have focused on the relationship between RT compliance and treatment outcomes in various types of malignancy [1-5]. These results suggested that failure to adhere to RT protocol guidelines compromises survival. Overall compliance of 92% in the current trial seemed acceptable to provide reliable results. More than half of the participating institutions did not have VU, and even with VU, the majority had only one VU; however, there is room for improving compliance in future trials incorporating RT. GTV and ENI violations and/or deviations were more frequent in the early period. In addition, among institutions with VU, the majority had VU in the first 3 cases. This may be because the institutions received feedback on how to better comply with the treatment protocol by the RT principal investigator, which enabled participants to follow the protocol guidelines in their later cases.

In the current study, more suboptimal treatments were observed in field placement than in the dose for tumors or risk organs. A similar trend was reported in other studies [7,8]. The majority of VU consisted of smaller lateral margins. The reason may have been a discrepancy between the protocol guidelines and their daily practices. The physicians tended to reduce lateral margins rather than craniospinal margins for fear of radiation pneumonitis. The varied ENI coverage also suggested a discrepancy. In this trial, a dry-run procedure was not attempted and therefore the radiation oncologists in each institution might not have been familiar with the protocol guidelines in the initial period of this trial. Wallner et al. [4] speculated the influence of clinical trial experience by reviewing a large number of cases in RTOG studies for lung and head and neck cancer. They reported that adequate primary and lymph node margins and dose prescriptions had progressively improved over the years, suggesting long-lasting learning experiences in clinical trials. As the need for immediate monitoring was described by Schaake-Koning et al. [9] from a quality control study in the EORTC lung cancer trial, some early interventions, such as a dry-run and immediate feedback before the start of treatment, will be more effective to improve compliance in clinical trials involving RT.

There were several limitations of our study. We did not perform 3-D volumetric data analyses due to technical limitations. Other factors, such as inter-observer contouring variations, 2-D vs. 3-D planning, may have had a much greater impact on the outcome of this trial than protocol compliance. The transition from 2-D to 3-D treatment planning is now almost complete in Japan, and more precise QA analyses using digital data, exported from treatment planning systems with the DICOM-RT format, have been introduced in recent JCOG 3-D RT trials.

In addition, all described QA activities focused on the medical aspects and treatment planning. Another important aspect is dosimetric QA. It is well known from the reports and scientific publications of the WHO/IAEA network [10], the ESTRO-EQUAL network in Europe [11] and the NCI network in the US [12] that external dosimetric audits are a powerful tool to avoid systematic errors. Dosimetric audits are generally recommended as integral parts of QA activities for clinical trials. In Japan, dosimetric audits were introduced in 2003, and were therefore not available at the beginning of this trial, and have been implemented in recent JCOG radiotherapy trials [13]. We also believe that these activities will have run-on effects in routine practice and lead to higher quality cancer care.

## Conclusion

In conclusion, the results of the RT QA assessment of JCOG 0202 seemed to be acceptable, providing scientifically reliable results. The time trend toward improved compliance in this trial showed the importance of introducing an RT QA program. A dry-run procedure and intensive feedback to participating institutions are being implemented to further improve JCOG trials.

## Competing interests

The authors declare that they have no competing interests.

## Authors' contributions

NS performed the QA evaluation. SI was in charge of the QA program and performed the QA evaluation. KH participated in the design of the QA program and helped to draft the manuscript. KK, and YN and TT conceived the study and helped to draft the manuscript.
